# Supplementary material for: Phytoplankton Size Structure and Diversity in the Transitional System of the Aquatina Lagoon (Southern Adriatic Sea, Mediterranean)
Source: Microorganisms. 2023 May 13;11(5):1277. doi: 10.3390/microorganisms11051277 (PMC10223948; doi:10.3390/microorganisms11051277)
Supplement: Supplementary file 1 [file microorganisms-11-01277-s001.zip › microorganisms-2348711-supplementary.pdf]

Table S1. List of the phytoplankton taxa identified in Aquatina Lagoon

**Bacillariophyceae**	**1996**	**2007**
Achnanthes adnata Bory		+
Amphora spp.		+
Bacteriastrum sp.		+
Biddulphia sp.	+	
Cerataulina pelagica (Cleve) Hendey		+
Chaetoceros curvisetus Cleve		+
Chaetoceros simplex Ostenf.		+
Chaetoceros spp.	+	+
Cocconeis scutellum Ehrenb.	+	+
Cocconeis sp.	+	+
Coscinodiscus granii L.F.Gough	+	
Coscinodiscus spp.	+	+
Cylindrotheca closterium (Ehrenb.) Reimann & J.C.Lewin	+	+
Cymatosira sp.		+
Grammatophora marina (Lyngb.) Kütz.		+
Guinardia delicatula (Cleve) Hasle		+
Halamphora coffeiformis (C.Agardh) Mereschkowsky	+	
Haslea wawrikae (Hust.) Simonsen		+
Leptocylindrus danicus complex		+
Licmophora flabellata (Greville) C.Agardh	+	
Licmophora gracilis var. *elongata* (Kütz.) De Toni		+
Licmophora gracilis (Ehrenb.) Grunow		+
Licmophora spp.	+	+
Lioloma pacificum (Cupp) Hasle		+
Melosira nummuloides C.Agardh	+	+
Navicula spp.	+	+
Nitzschia linearis W.Smith	+	
Nitzschia longissima (Bréb.) Ralfs	+	+
Nitzschia sigma (Kützing) W.Smith	+	
Nitzschia thermalis (Ehrenberg) Auerswald	+	
Nitzschia spp.	+	+
Pleurosigma elongatum C.W.Sm.		+
Pleurosigma spp.	+	+
Pseudo-nitzschia cf. *prolongatoides* (Hasle) Hasle		+
Pseudo-nitzschia spp.		+
Skeletonema sp.	+	
Tabellaria sp.	+	
Tetramphora ostrearia (Brébisson) Mereschkowsky	+	
Thalassionema bacillare (Heiden) Kolbe	+	+
Thalassiosira spp.		+
Undetermined centric diatoms	+	+
Undetermined pennate diatoms	+	

Table S1. List of the phytoplankton taxa identified in Aquatina Lagoon

	1996	2007
**Dinophyceae**		
Akashiwo sanguinea (K.Hirasaka) Gert Hansen & Moestrup	+	+
Alexandrium minutum complex		+
Alexandrium spp.		+
Amphidinium acutissimum J.Schiller	+	
Amphidinium curvatum J.Schiller	+	
Amphidinium sp.	+	
Dinophysis sacculus F.Stein	+	+
Dinophysis sp.	+	
Dinopyxis compressa (Bailey) F.Stein		+
Diplopsalis spp.		+
Gonyaulax spinifera (Clap. & J.Lachm.) Diesing	+	+
Gonyaulax spp.	+	+
Gymnodinium marinum Kent		+
Gymnodinium spp.	+	+
Gyrodinium fusiforme Kof. & Swezy		+
Gyrodinium spp.		+
Heterocapsa niei (A.R.Loebl.) L.C.Morrill & A.R.Loebl.		+
Kryptoperidinium triquetrum (Ehrenb.) Tillmann, Gottschling, Elbr., Kusber & Hoppenrath		+
Lebouridinium glaucum (M.Lebour) F.Gómez H.Takay, D.Moreira & P.López-García		+
Noctiluca scintillans (Macartney) Kof. & Swezy		+
Prorocentrum bidens J.Schiller	+	
Prorocentrum cordatum (Ostenf.) J.D.Dodge	+	+
Prorocentrum micans Ehrenb.	+	+
Prorocentrum triestinum J.Schiller	+	+
Protoperidinium spp.	+	+
Scrippsiella acuminata (Ehrenb.) Kretschmann, Elbr., Zinssm., Soehner, Kirsch, Kusber & Gottschling	+	+
Scrippsiella spp.	+	+
Torodinium teredo (C.H.G. Pouchet) Kof. & Swezy		+
Undetermined naked dinoflagellates	+	
undetermined thecate dinoflagellates	+	
**Prymnesiophyceae**		
Emiliania huxleyi (Lohmann) W.W.Hay & H.P.Mohler		+
Undetermined coccolitophores		+

Table S1. List of the phytoplankton taxa identified in Aquatina Lagoon

**Other phytoplankton**	**1996**	**2007**
Euglena sp.	+	+
Eutreptia viridis Perty		+
Eutreptiella marina A.M. Cunha		+
Leptolyngbya sp.		+
Mesodinium rubrum (Lohmann) Leegard	+	
Oscillatoria sp.		+
Synechococcus/Cyanobium type		+
Xenococcus schousboei Thuret	+	
Undetermined Euglenophyceae	+	+
Undetermined Cyanobacteria	+	+
Undetermined phytoflagellates	+	+

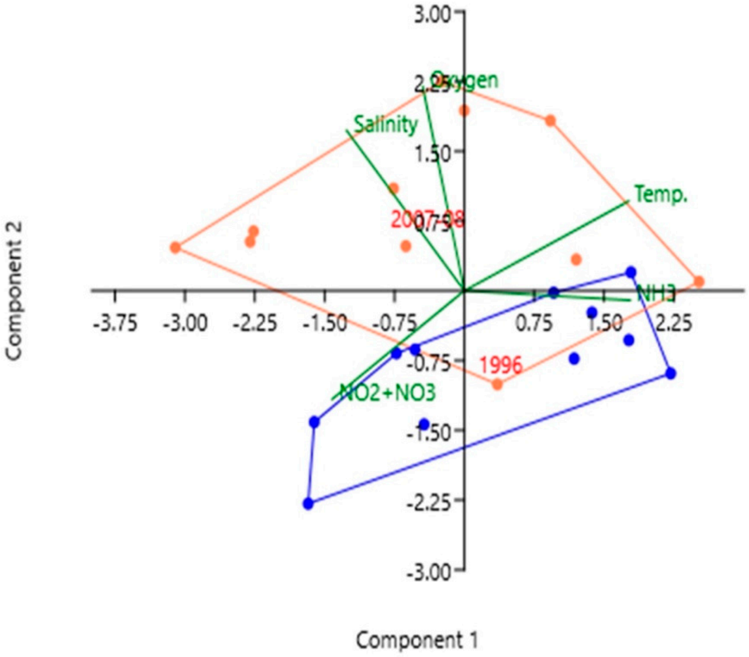

Figure S1. Principal Component Analysis of the environmental values observed in the Aquatina Lagoon during the periods January 1996 - December 1996 and April 2007 - March 2008.
